# Supplementary material for: Synergistic reduction of graphene oxide using vitamin C and urea: Enhanced efficiency and material properties
Source: PLoS One. 2025 Sep 4;20(9):e0330990. doi: 10.1371/journal.pone.0330990 (PMC12410778; doi:10.1371/journal.pone.0330990)
Supplement: S1 Table — (DOCX) [file pone.0330990.s001.docx]

S1Table. Main experimental reagents

| **Reagent Name** | **Model/Specification** | **Purity** | **Manufacturer** |
| --- | --- | --- | --- |
| Graphite Powder | CP | ≥99.8%  （~200 μm） | Qingdao Dongkai Graphite Co., Ltd. |
| Ethyl Alcohol | CP | 95% | Sinopharm Chemical Reagent Co., Ltd. |
| KMnO_4_ | AR | ≥99.3% | Sinopharm Chemical Reagent Co., Ltd. |
| H_2_SO_4_ | CP | 95-98% | Sinopharm Chemical Reagent Co., Ltd. |
| H₂O₂ | AR | 30% w/w | Sinopharm Chemical Reagent Co., Ltd. |
| HCl | CP | 36-38% | Sinopharm Chemical Reagent Co., Ltd. |
| Vitamin C(VC) | AR | ≥99.0% | Sinopharm Chemical Reagent Co., Ltd. |
| Urea | AR | ≥99.5% | Sinopharm Chemical Reagent Co., Ltd. |
