# Supplementary material for: Synergistic reduction of graphene oxide using vitamin C and urea: Enhanced efficiency and material properties
Source: PLoS One. 2025 Sep 4;20(9):e0330990. doi: 10.1371/journal.pone.0330990 (PMC12410778; doi:10.1371/journal.pone.0330990)
Supplement: Attachment — Submitted filename: Response to Reviewers.pdf [file pone.0330990.s003.pdf]

Synergistic Reduction of Graphene Oxide Using Vitamin C and Urea: Enhanced Efficiency and Material Properties

Dear reviewers：

Thank you very much for your patience in reading my paper and giving me a number of meaningful suggestions. I have read it carefully and put forward corresponding revisions according to your suggestions, my revisions are as follows, and I am honored to have your review.

Reviewer’s comments	Answer to reviewer’s comments
Reviewer 1	
1.Language and Grammar:<br>The manuscript contains numerous grammatical and syntactical errors that hinder readability. A thorough revision by a native English speaker or professional editing service is strongly recommended. Examples:<br>• Pag 17 line 11: “more order” → “more ordered”	（1）Thank you for your suggestion. We have addressed grammatical and syntactic shortcomings through targeted revisions.<br>（2）Following Reviewer 1 and 2's recommendation, we changed "more order" to "showed broadened peaks" on Page 17, Line 11.
2.Formatting Errors:<br>• Equations (1) – (5) are not properly formatted or numbered in the text.<br>• There is a persistent formatting issue: “Error! Reference source not found.” in the BET section. This must be corrected.	（1）We have numbered Equations (1) - (5).<br>（2）Reference [49] has been replaced in the revised manuscript.
3. Experimental Details	(1) In Section 2.2 and subsequent figures, we have added the exact mass ratios

The manuscript lacks critical experimental parameters:<br>• Exact VC/GO and Urea/GO mass ratios. Mass ratios for rGO-VC and rGO-Urea are omitted in Section 2.2. For rGO-VC+Urea, "mass ratio of rGO(10):Vc(0.5):Urea(10)" is confusing (rGO is the product). Specify as GO:VC:urea = 10:0.5:10.<br>• pH of the reaction mixtures.<br>• Details on the reproducibility of the synthesis (e.g., number of replicates).	for synthesizing rGO.<br>(2) Thank you for carefully identifying this error. For rGO-VC+Urea, the description "mass ratio of rGO(10):Vc(0.5):Urea(10)" was confusing (rGO is the product). This has been revised to "GO:VC:urea = 10:0.5:10" in the manuscript.<br>(3) Since the reaction occurred in a sealed self-pressurized reactor, urea decomposition during the process led to dynamic pH changes. The pH of the reaction mixture was not measured, but the post-reaction solution was alkaline.<br>(4) Repeated experimental verification has been added to the "rGO preparation" section of the Methods：All experiments were done in two or three replicates.
4.Data Presentation<br>Abstract/Results: Claims "superior electrical conductivity (1.94 Ω)" are incorrect.<br>• Ω (ohm) measures resistance, not conductivity (measured in S/cm or S/m).<br>Table 1 Issues:<br>• Units for "rGO conductivity" are mixed (Ω, $S{\cdot}cm^{-1}$, $F{\cdot}g^{-1}$). Include a column for metric type (e.g., resistance, conductivity, capacitance).<br>• The BET data should be summarized in a table with surface	(1) We sincerely thank you for identifying the error in electrical parameter reporting. The phrase "superior electrical conductivity (1.94 Ω)" has been corrected to "resistivity ρ = 1.2 Ω • cm" throughout the Abstract and Results. We confirm the inverse relationship: conductivity σ = 1/ρ (where 1 S/cm = 1 $Ω^{-1}$ • cm^{-1}).<br>(2) The manuscript title now explicitly includes "resistance, conductivity, capacitance" to better reflect the studied properties.<br>(3) Average pore size and specific surface area data have been consolidated into Table 1.

area, pore volume, and average pore size.	
5. Discussion<br>• The proposed mechanism is interesting but speculative. The authors should support their claims with additional evidence (e.g., XPS for nitrogen doping).<br>• The role of urea in creating an alkaline environment and its interaction with VC is plausible but needs clearer chemical justification.	(1) We appreciate your suggestion regarding XPS analysis. While XPS could provide direct evidence of nitrogen doping, our alternative characterizations and prior literature support reasonable inference of N-doping. Due to equipment limitations, XPS was not performed, and we hope this explanation clarifies our constraints.<br>(2) We provide the following chemical justification for urea's role:<br>Urea Decomposition:Urea thermally decomposes to ammonia , creating an alkaline environment.<br>Alkaline-Mediated VC Enhancement: This alkaline milieu elevates VC's nucleophilicity through deprotonation of its enediol group.<br>Synergistic Nucleophilic Attack: Both activated VC and urea-derived species concurrently attack electrophilic sites (e.g., epoxides, carbonyls) on GO.<br>(3) Mechanistic Validation: These reaction pathways are systematically analyzed in Section X: Reduction Mechanisms Exploration.
6. References:<br>• Ref 33 (Ren et al., 2025) is post-submission (2025). Verify accessibility/cite as "in press" if applicable.<br>• Ref 21: Author list truncated ("B.Lesiak, et al.").<br>• Some inconsistencies in formatting and citation numbering.	(1) Reference 33 is now accessible for download in the SCI database.<br>(2) Reference 21 has been reformatted according to standard citation guidelines.<br>(3) All references have been thoroughly reorganized based on the reviewer's suggestions, with additional new references incorporated.

Reviewer 2	
1. In the Materials and Chemicals section, please list all the chemicals used along with their respective purities.	A comprehensive list of all chemical reagents used in this study has been provided as S1 Table in the Supporting Information.
2.In the rGO Preparation section, clearly specify the mass ratios of the reagents, reaction temperatures, and durations involved in the synthesis process.	In the rGO reaction studies, various parameters were examined including reagent mass ratios, reaction temperatures, and durations. As each experiment had unique conditions, it was not feasible to specify all reaction parameters in the Experimental section. Instead:<br>Common procedures (experimental steps and chemical dosages) are described in the main text<br>Specific reaction conditions for each test are provided in the corresponding figure captions
3. There is a lack of experimental data for the rGO-VC+Urea sample in terms of resistivity and resistance. Please include more rGO-VC+Urea samples to allow proper comparison with the rGO-VC and rGO-Urea samples.	As shown in the newly added Fig. 1(d) (120° C, 2h reaction):<br>Comparative analysis of different reductant/GO ratios confirms that the VC-urea co-reductant system significantly enhances rGO performance，particularly in reducing electrical resistivity and resistance.
4. The Y-axis title is missing in the XRD figure.	We thank the reviewer for this suggestion. The requested additions have been incorporated into the revised manuscript.
5.The phrase “more order” in the statement “Compared to rGO-Urea, the XRD peaks of rGO-VC and rGO-VC+Urea are more order” is unclear. Please elaborate on what is meant by “more order” — for example, whether it refers to higher	Yes, thank you for the correction. Following your suggestion and to best convey our findings, we have revised the text to state: "rGO-VC and rGO-VC+Urea exhibit broader XRD peaks than rGO-Urea."

crystallinity, or sharper peaks.	
6. Remove the text “Error! Reference source not found.” in the BET analysis section and provide the correct reference to support the statement.	Reference [49] has been replaced in the revised manuscript.
7. In Table 3, please provide appropriate references for Urea, VC, and VC+Urea. For samples obtained in the current work, label them as “this study.”	We thank the reviewer. The phrase "this study" has been added as suggested.
Reviewer 3	
1. In Introduction, authors should add a Table that compares the rGO, preparation methods and physicochemical characteristics with published literatures?	In the Introduction, we:<br>(1) Discussed various graphene preparation methods,<br>(2) Described different reductants for GO-to-rGO conversion,<br>(3) Specifically summarized VC/Urea-based GO reduction studies in Table 1.<br>We have further improved both the Introduction and Table 1. Given this comprehensive coverage, we believe a separate comparative table would be redundant. We hope this clarifies our rationale.
2. On what basis do authors decide to use vitamin C and urea for reduction of GO?	In the revised Introduction, we have added explanations for selecting VC and urea as GO reductants, based on three key considerations:<br>（1）Accessibility and Safety: VC and urea are readily available and exhibit high safety profiles.<br>（2）Dual Function of Urea:<br>- Reduces GO

	- Decomposes to create an alkaline environment<br>- Enhances VC’s nucleophilicity → Improves VC’s reducing capability<br>（3）Gas-Mediated Expansion:<br>- Gaseous products from urea decomposition promote interlayer expansion of graphene<br>- Increase interlayer spacing → Enhance reductant diffusion between GO sheets<br>These synergistic mechanisms motivated our experimental investigation of their combined effects.
3. In Introduction, authors should explain deeply what is the novelty of this study?	In the revised Introduction, we:<br>(1) Cite Galvão et al. and Jian Gao et al. to establish the viability of co-reductant systems for GO reduction.<br>(2) Specifically demonstrate urea's dual role:<br>- Direct reduction of GO<br>- Generation of an alkaline environment (via ammonia decomposition) that enhances VC's nucleophilicity, thereby facilitating GO reduction.<br>(3) Explicitly state:<br>"The investigation of this synergistic mechanism between VC and urea constitutes the key innovation of this study."
4.In Introduction section, authors should add a short paragraph on characteristics of graphene oxide (GO).	The suggested additions have been incorporated into the revised manuscript.

5. In rGO Preparation, authors should correct the spelling “Preparation”? What is the impact of ultrasonication on GO?	(1) Thank you for your suggestion. We sincerely apologize for the typographical error and have corrected it.<br>(2) Ultrasonication enhances both the speed and uniformity of GO dispersion in water. This method is commonly employed in prior studies as an auxiliary technique.
6. In BET analysis, authors should explain why the surface area is highest for rGO prepared from combination of VC and urea?	When both agents are used simultaneously, a broader spectrum of oxygen-containing functional groups is removed more thoroughly. This leads to a significant reduction in polar groups on the graphene sheets and a marked weakening of the strong interlayer hydrogen bonding interactions. The presence of ascorbic acid (VC) accelerates the reduction kinetics. It is plausible that this acceleration results in the partial reduction of the sheets and imparts sufficient flexibility by the time gaseous products from urea decomposition are generated. This flexible, partially reduced state allows the sheets to be more readily expanded by the evolving bubbles, facilitating the formation of a porous structure. Conversely, when urea is used alone, the lower reduction efficiency likely leaves the sheet structure insufficiently "open" or exfoliated to effectively utilize the bubbling action for pore creation. Urea, however, contributes significantly to dispersion stability through physisorption and by establishing a hydrogen bonding network, providing enhanced spatial steric hindrance.
7. In XRD spectra, two peaks around 11° and 13° can be	In rGO-Urea samples, two minor characteristic GO peaks observed at 11° and

observed for rGO prepared from urea, what are these peaks?	13° indicate incomplete reduction of the product.
8. Authors may shift main figures into the manuscript instead of presenting them in supplementary information.	The main figures have now been integrated directly into the manuscript text to facilitate your review.
