# Supplementary material for: Synergistic reduction of graphene oxide using vitamin C and urea: Enhanced efficiency and material properties
Source: PLoS One. 2025 Sep 4;20(9):e0330990. doi: 10.1371/journal.pone.0330990 (PMC12410778; doi:10.1371/journal.pone.0330990)
Supplement: Attachment — Submitted filename: Review Comments.docx [file pone.0330990.s002.docx]

The authors in the present manuscript show the rGO (rGO-VC+Urea) was prepared by combining vitamin C (VC) and urea as co-reducing agents with the modified Hummer’s method. Compared to samples reduced solely with VC or urea, the co-reducing agents significantly reduced the required reaction time (to 2 hours) and temperature (to 120°C), while yielding material with superior electrical conductivity (1.94 Ω). The structure of the samples was characterized using XRD, FT-IR, Raman spectroscopy, BET surface area analysis, and SEM. Results indicate that the sample prepared from co-reducing agents possesses a typical graphene structure and incorporates C-N bonds. The authors should address the following issues and information’s before publication acceptance in the prestigious ‘PLOS One’ Journal:

1. In Introduction, authors should add a Table that compares the rGO, preparation methods and physicochemical characteristics with published literatures?

2. On what basis do authors decide to use vitamin C and urea for reduction of GO?

3. In Introduction, authors should explain deeply what is the novelty of this study?

4. In Introduction section, authors should add a short paragraph on characteristics of graphene oxide (GO). Authors may go through these publications for more details and cite accordingly: <https://doi.org/10.1016/j.cartre.2023.100251> & <https://doi.org/10.1016/j.carbon.2024.119331>

5. In rGO Preparation, authors should correct the spelling “Preparation”? What is the impact of ultrasonication on GO?

6. In BET analysis, authors should explain why the surface area is highest for rGO prepared from combination of VC and urea?

7. In XRD spectra, two peaks around 11° and 13° can be observed for rGO prepared from urea, what are these peaks?

8. Authors may shift main figures into the manuscript instead of presenting them in supplementary information.
